# Nanosilica enhances morphogenic and chemical parameters of M*egathyrsus maximus* grass under conditions of phosphorus deficiency and excess stress in different soils

**DOI:** 10.1186/s12870-023-04521-3

**Published:** 2023-10-17

**Authors:** Cíntia Cármen de Faria Melo, Danilo Silva Amaral, Anderson de Moura Zanine, Daniele de Jesus Ferreira, Renato de Mello Prado, Marisa de Cássia Piccolo

**Affiliations:** 1 Laboratory of Plant Nutrition, Department of Agricultural Production Sciences (Soil and Fertilizer Sector), School of Agricultural and Veterinarian Sciences, São Paulo State University (UNESP), Prof. Paulo Donato Castellane Avenue, Jaboticabal, SP 14884900 Brazil; 2 Department of Engineering and Exact Sciences, School of Agricultural and Veterinarian Sciences, São Paulo State University (UNESP), Prof. Paulo Donato Castellane Avenue, Jaboticabal, SP 14884900 Brazil; 3 Center for Agricultural and Environmental Sciences, Department of Animal Science, Federal University of Maranhão, BR 222 km 04 Highway, Chapadinha, MA 65500000 Brazil; 4 Laboratory of Nutrient Cycling, Center of Nuclear Energy in Agriculture, University of São Paulo (USP), 303 Centenário Avenue, Piracicaba, SP 13400970 Brazil

**Keywords:** Silicon fertigation, Zuri guinea grass, Phosphorus nutritional stresses, Lignin, Tillering

## Abstract

Phosphorus (P) imbalances are a recurring issue in cultivated soils with pastures across diverse regions. In addition to P deficiency, the prevalence of excess P in soil has escalated, resulting in damage to pasture yield. In response to this reality, there is a need for well-considered strategies, such as the application of silicon (Si), a known element for alleviating plant stress. However, the influence of Si on the morphogenetic and chemical attributes of forage grasses grown in various soils remains uncertain. Consequently, this study aimed to assess the impact of P deficiency and excess on morphogenetic and chemical parameters, as well as digestibility, in Zuri guinea grass cultivated in Oxisol and Entisol soils. It also sought to determine whether fertigation with nanosilica could mitigate the detrimental effects of these nutritional stresses. Results revealed that P deficiency led to a reduction in tiller numbers and grass protein content, along with an increase in lignin content. Conversely, P excess resulted in higher proportions of dead material and lignin, a reduced mass leaf: stem ratio in plants, and a decrease in dry matter (DM) yield. Fertigation with Si improved tillering and protein content in deficient plants. In the case of P excess, Si reduced tiller mortality and lignin content, increased the mass leaf:stem ratio, and enhanced DM yield. This approach also increased yields in plants with sufficient P levels without affecting grass digestibility. Thus, Si utilization holds promise for enhancing the growth and chemical characteristics of forage grasses under P stress and optimizing yield in well-nourished, adapted plants, promoting more sustainable pasture yields.

## Background

Yield in pastoral ecosystems worldwide is susceptible to phosphorus (P) limitation, particularly in tropical regions where deficiencies are prevalent [1]. In these regions, extensive weathering, and the high phosphate adsorption capacity of soils diminish nutrient availability, particularly in clayey soils [2]. This necessitates phosphate fertilization to sustainably boost forage yield [3]. Conversely, in intensive agriculture, the frequent application of high doses of phosphorus (P) fertilizers, whether conventional or organic, can result in excessive P accumulation within plants, surpassing the quantities exported from the fields [4]. This leads to nutritional imbalances and an overabundance of P within plant tissues [5, 6]. The risk of P excess is further exacerbated in soils with a sandy texture (clay content < 15%), where phosphate availability is heightened due to relatively low element adsorption in the soil [2].

Within plants, the cellular balance of inorganic phosphate (Pi) plays a fundamental role in converting nutrients such as carbon (C) and nitrogen (N) into biomass, as P serves vital metabolic and structural functions [5, 7]. Phosphorus imbalances compromise plant physiological processes [8], consequently affecting the morphogenic processes that determine forage growth. For instance, disruptions in P levels within the plant can diminish leaf and stem appearance and elongation [9, 10], as well as plant tillering [11–13], ultimately reducing forage yield and pasture sustainability.

The repercussions of P imbalances within plants extend beyond growth rates and biomass yield to influence forage quality. Forage grasses encompass carbohydrates, proteins, lipids, fibers, lignin, minerals, and vitamins, all of which significantly impact herd productivity [14]. Nutritional imbalances can alter these components, potentially affecting the nutritional value of the pasture [15, 16]. However, further research is needed to comprehensively understand these effects.

Given the global prevalence of P imbalances in plants [1, 4], there is an urgent need for strategies to mitigate these losses. Research efforts have been directed toward finding more efficient and sustainable methods for phosphate nutrition in forage plants [17]. One emerging option for addressing these imbalances involves silicon (Si), with initial reports suggesting promise in mitigating nutritional deficiencies [18]. However, these studies have primarily focused on soilless hydroponic cultivation [19, 20], leaving the role of Si in the morphogenetic responses of grasses under P deficiency when cultivated in soil unexplored. Moreover, the effects of excess P on grasses remain largely unknown, including its impact on plant morphogenesis, necessitating further investigation. It is conceivable that Si may also contribute to alleviating excess P, given its known benefits to this plant group (Poaceae) when subjected to other nutritional stresses [21, 22].

It is important to note that the potential benefits of Si in stress mitigation hinge on the plant’s high uptake of the element, which, in turn, depends on the source used. Recent advancements in nanotechnology have introduced the option of nanoparticulate silicon [23], which holds the potential to enhance Si uptake due to the monomeric size of SiO_2_ particles that remain stable in solution [24, 25]. Consequently, there is an expectation that employing this Si source may offer promising prospects for mitigating nutritional stress, warranting further investigation.

To shed light on the effects of P imbalances in forage grasses and the potential benefits of Si in alleviating these stresses, it is crucial to address the following questions: (i) How does damage resulting from P deficiency and excess affect the morphogenesis of *Megathyrsus maximus* cv. Zuri grass and impede P yield? (ii) Does it influence chemical parameters and the digestibility of plant biomass? (iii) Is nanosilica fertilization an efficient strategy? In other words, does it enhance Si uptake by the plant, thereby mitigating the damage caused by P deficiency and excess through improvements in plant morphogenesis and forage quality when cultivated in both clayey and sandy soils?

If these hypotheses are substantiated, it will mark the first comprehensive understanding of how this forage grass species responds to morphogenic changes when subjected to P deficiency or excess and how this affects forage quality. Furthermore, it may indicate the utility of employing nanoparticulate Si in forage cultivation to ameliorate issues related to P deficiency or excess in soils of varying textures, thereby bolstering the sustainable cultivation of this species in regions grappling with P imbalances.

## Methods

### Experimental design and cultivation environment

Two experiments were conducted with *Megathyrsus maximus* cv. Zuri, cultivated in Oxisol (Experiment 1) and Entisol (Quartzipsamment) (Experiment 2) [26] under greenhouse conditions. These experiments were carried out at São Paulo State University, Jaboticabal, Brazil, spanning from October 2021 to April 2022. Throughout this period, six defoliation intervals were imposed, corresponding to six growth cycles. Cultivation occurred under full natural light conditions. Inside the greenhouse, the average maximum air temperature reached 47 ± 5 °C, while the average minimum temperature was 22 ± 5 °C, with an average relative humidity of 50 ± 10%.

The soils were collected from the surface layer of the uncultivated area, in the 0 to 20 cm layer, and the samples were air-dried and passed through a 6-mm mesh sieve. Both experiments followed a 3 × 2 factorial design, encompassing three phosphorus nutritional states: deficient (no P application), sufficient (200 mg dm^− 3^ of P) [27], and excessive (600 mg dm^− 3^ of P). These states were combined with the absence of silicon (Si) application (no Si) and the presence of Si (1.5 mM Si solution) [28]. The experiments were arranged in a completely randomized design with four replications. Each experimental unit consisted of a plastic pot with a volume of 7.2 L (dimensions: height 32 cm, bottom base 14 × 14 cm, and top 16 × 16 cm), filled with Oxisol or Entisol samples, each pot containing two plants.

### Characterization of soils and application of treatments

Soil samples were collected from native forests and subsequently subjected to chemical analysis following established procedures [29]. The results for Oxisol and Entisol, respectively, were as follows: pH CaCl_2_ = 3.8 and 4.3; organic matter (OM) = 34 and 9.0 mg dm^− 3^; P in resin extractor = 12 and 2 mg dm^− 3^; K = 1.4 and 3.0; Ca = 6 and 3.0; Mg = 2 and 1.0 mmolc dm^− 3^; H + Al = 85 and 16; and cation exchange capacity (CEC) = 94 and 20.3 mmolc dm^− 3^. Silicon contents were determined [30], yielding the following outcomes: 3.0 and 1.0 g dm^− 3^ for Oxisol and Entisol, respectively. Granulometric analysis, conducted according to [31], provided the following results for Oxisol and Entisol, respectively: 51 and 94% sand, 6 and 1% silt, and 43 and 5% clay, with texture [32] sandy clay (Oxisol) and sand (Entisol).

To address soil requirements, liming was carried out using limestone with a total neutralization power of 125%, consisting of CaO (58.5%) and MgO (9%), to correct acidity and increase base saturation to 70% [29]. Forty days after liming, phosphorus (P) was incorporated into the soil at varying rates: 0, 200 [27], and 600 mg dm^− 3^ of P, administered in the form of triple superphosphate, corresponding to the deficiency, sufficiency, and excess P treatments, respectively. Additionally, 225, 50, 5, and 0.5 mg dm^− 3^ of K, S, Zn, and B were applied to both soil types. In Entisol, an additional 5 mg dm^− 3^ of Fe was required, supplied through potassium chloride (52.4% K), calcium sulfate dihydrate (18% S), zinc sulfate heptahydrate (35% Zn), boric acid (17% H_3_BO_3_), and iron chelate (EDDHA) (6% Fe) sources, respectively. Nitrogen (300 mg dm^− 3^) was provided incrementally during each regrowth period in the form of urea (45% N), with subsequent irrigation.

Sowing was directly performed in the soil using seeds from the Zuri grass cultivar, developed by the Brazilian Agricultural Research Corporation and commercially acquired. Each pot contained two plants, which underwent a standardization cut at 17 cm above ground level 30 days after sowing, marking the commencement of the data collection phase. In the case of the treatment deficient in Entisol (0 mg dm^− 3^ of P), an additional 4 mg dm^− 3^ of P was applied on the day of standardization to facilitate minimal plant growth, whereas Oxisol did not necessitate such intervention for minimal plant development. Irrigation management was conducted to maintain soil water retention at 70%, monitored by daily pot weighing and compensating for water losses due to evapotranspiration [33, 34].

Silicon application occurred daily from the sowing date, utilizing a 1.5 mmol L^− 1^ Si solution [28]. Colloidal nanosilica served as the Si source, characterized by particle sizes ranging from 8.5 to 9.7 nm, a specific surface area of 300 m^2^ g^− 1^, and a pH of 10.5. The same volume of solution was applied across all treatments, with the daily volume determined based on the treatment with the lowest water demand. On average, 102 and 69 mg dm^− 3^ of Si were administered during each forage regrowth in Oxisol and Entisol, respectively. In treatments without Si application, irrigation was performed using deionized water. Cuts were executed when plants under P sufficiency reached a height of 70 cm, leaving a residual biomass of 30 cm in height [35].

### Variables analysed

#### Morphogenic and structural variables

Growth monitoring encompassed six regrowth cycles of the plants. In each regrowth phase, two tillers per pot were meticulously selected and distinguished with colored ribbons. These tillers were examined every three days, focusing on the exposure of ligules, stem length, and the length of leaf blades in various states: expansion, expanded, or undergoing senescence. At the end of each growth cycle, the following parameters were assessed immediately before plant cutting: number of leaves per tiller, number of tillers per pot, count of live and dead leaves, and tiller diameter, with measurements performed using a digital caliper.

Following data collection, the subsequent variables were computed in accordance with [10]: leaf appearance rate (LA, leaves per tiller per day), leaf elongation rate (LE, measured in millimeters per day), number of live leaves per tiller, stem elongation rate (SE, expressed in millimeters day^− 1^), and stem diameter (SD, measured in millimeters). Tillering was also evaluated, considering the following parameters: number of tillers per pot, tiller appearance rate (TA = number of new tillers per total tillers alive in the previous cycle)/days of regrowth, tiller mortality rate (TM = number of dead tillers per total tillers alive in the previous cycle)/number of regrowth days, and tiller survival rate (TS = 1-TM) [36, 37].

#### Yield and morphological fractions

The average dry matter (DM) yield was determined through six collections of biomass from the plant’s grazing stratum [35]. In each collection, the morphological components, including leaf blades, stems, and dead material, were isolated from the biomass and measured in grams of fresh weight. This material was then placed in paper bags and subjected to drying in an oven with forced air circulation (maintained at 65 ± 5 ºC) until a constant mass was achieved. Subsequently, the dry matter (DM) content of the leaf blades, stems, and dead material was determined. Finally, the percentage composition of each morphological component, the mass leaf blade:stem ratio, and the total DM yield (in grams per pot) were calculated.

#### P and Si contents

P and Si contents in DM of the grazing stratum were determined. To determine P, the sample was subjected to nitric-perchloric digestion, with a spectrophotometer reading at 420 nm [38]. Si extraction was carried out by alkaline digestion in hydrogen peroxide at 120ºC [39], and determined by spectrophotometer reading at 410 nm after colorimetric reaction with ammonium molybdate [30]. P and Si contents were expressed in g kg^− 1^.

#### Chemical composition of forage

The processed material underwent milling using a Willey-type mill, enabling the determination of percentages for neutral detergent fiber (NDF), acid detergent fiber (ADF), and lignin in the dry mass [40, 41]. This data was used to calculate hemicellulose (the difference between NDF and ADF) and cellulose (the difference between ADF and lignin) contents. Additionally, the percentages of mineral matter (MM), non-fiber carbohydrates (NFCs), and crude protein (PB% = N content x 6.25, with N determined using a LECO Truspec CHNS analyzer) (AOAC, 2012) were computed. In vitro dry matter digestibility (IVDM) was also assessed [42]. The following equation was employed to estimate total digestible nutrients (TDN%): TDN (%) = Deg + (1.25*EE) – MM, where Deg represents degradability, 1.25 is the correction factor, EE signifies ether extract, and MM denotes mineral matter.

### Statistical analyses

The data were subjected to assessments of normality, homogeneity, and the independence of residuals [43, 44]. Subsequently, analysis of variance was conducted using the F test (with p < 0.05). In cases where significant differences among means were detected, Tukey’s post hoc test (p < 0.05) was employed for comparisons. These statistical analyses were carried out using the SPEED Stat version 2.8 software [45].

## Results

### Phosphorus and silicon contents

**Fig. 1:**
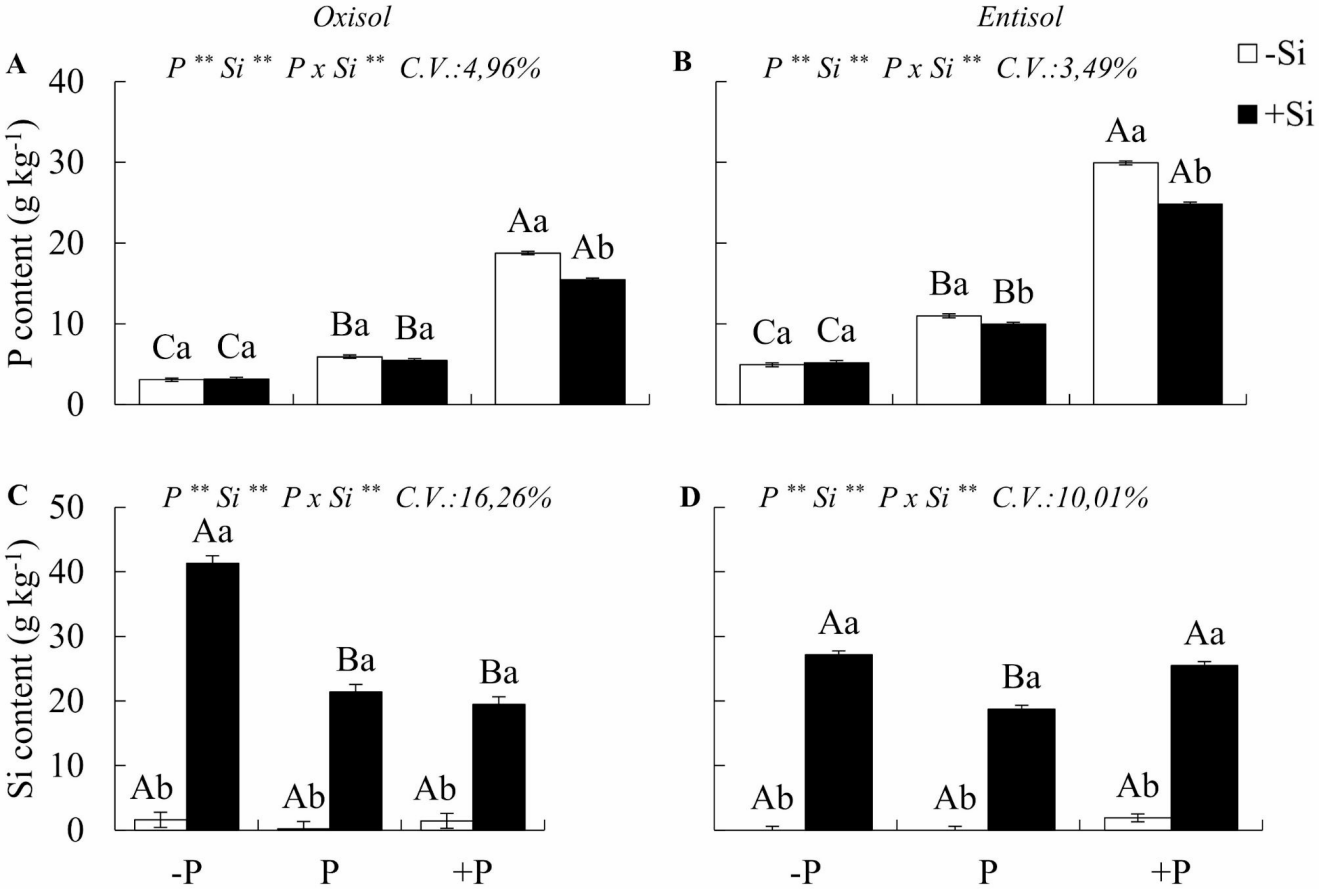
Phosphorus content and silicon content of Zuri guinea grass cultivated under different phosphorus levels of deficiency (- P), adequacy (P), and excess (+ P) in Oxisol (**A, C**) and Entisol (**B, D**) in absence (-Si) and presence of silicon (+ Si, 1.5 mmol L^− 1^). Ns, *, ** refer to F test not significant, significant at 5 and 1%, respectively. Nutritional states are compared in uppercase letters, and the effect of Si in lowercase letters (Tukey test, p < 0.05). Bars correspond to the standard error of the experiment mean

A significant interaction PxSi (p < 0.05) was observed for P and Si contents in plants. In the absence of Si, the deficiency and excess of P in the soil in relation to the adequate state caused a decrease and increase in the P content of the plant, respectively, which occurred in both soils (Fig. 1a, b), without changes in the levels of Si independent of the P state (Fig. 1c, d).

In the presence of Si, the P content was reduced in plants under excess P in both soils, and decreased in plants under adequate P status in Entisol, but there was no change in the P content of deficient plants when they received Si (Fig. 1a, b). Furthermore, the Si content increased in plants across all P states in both soils when they received nanosilica fertilization (Fig. 1c, d). The Si content was higher in plants deficient in P in Oxisol (Fig. 1c) and in P deficiency and excess in Entisol (Fig. 1d).

### Morphogenic and structural characteristics

**Fig. 2:**
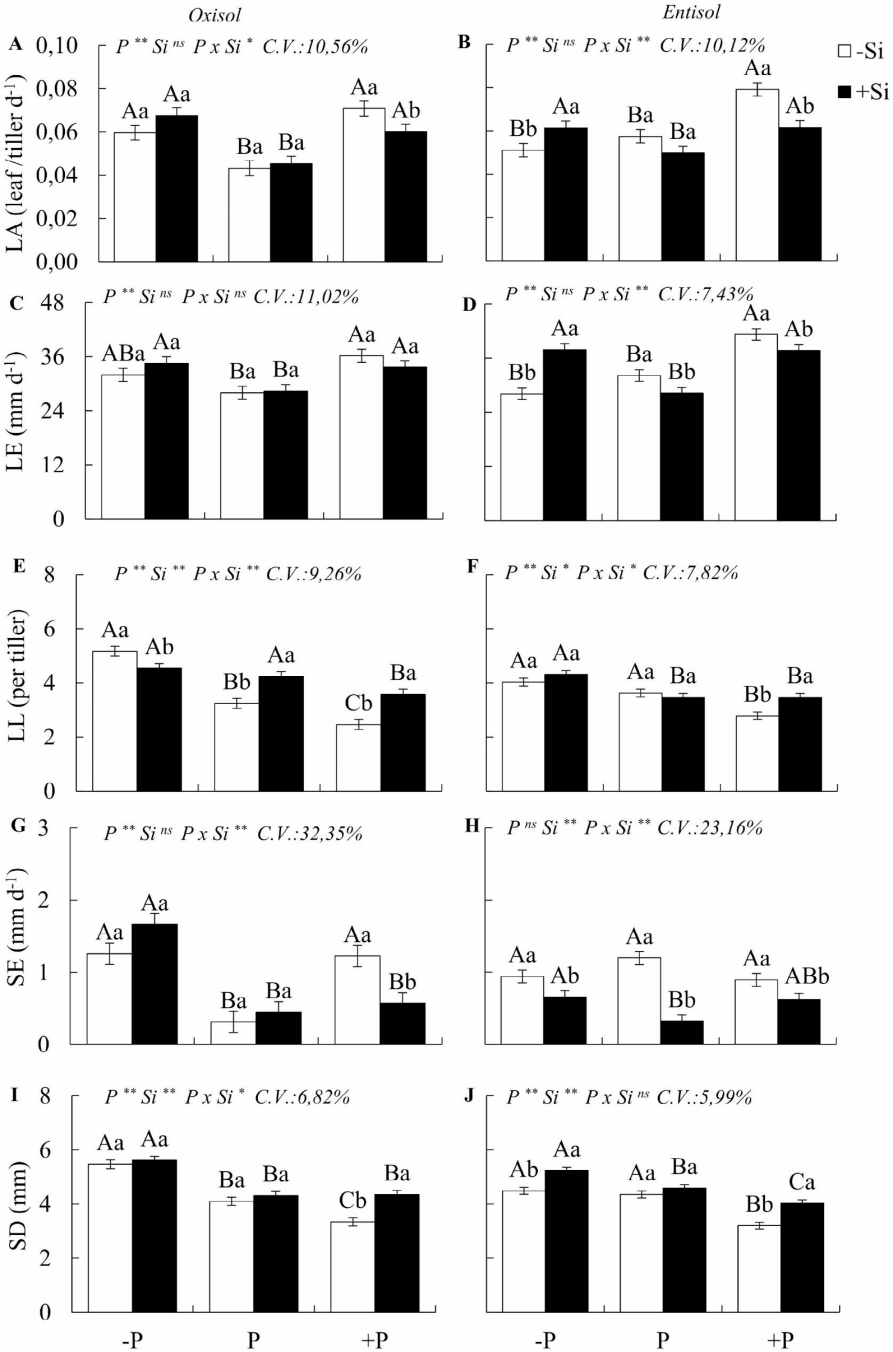
Leaf appearance rate (LA), leaf elongation rate (LE), number of live leaves per tiller (LL), stem elongation rate (SE), and stem diameter (SD) of Zuri guinea grass under different phosphorus levels deficiency (-P), sufficiency (P), and excess (+ P) in Oxisol (A, C, E, G, and I) and Entisol (B, D, F, H, and J) in the absence (-Si) and presence of silicon (+ Si, 1.5 mmol L^− 1^). Ns, *, ** denote the F test results as not significant and significant at 5 and 1%, respectively. Nutritional states are compared using uppercase letters, and the effect of Si is compared in lowercase letters (Tukey test, p < 0.05). Bars represent to the standard error of the experiment mean

**Fig. 3:**
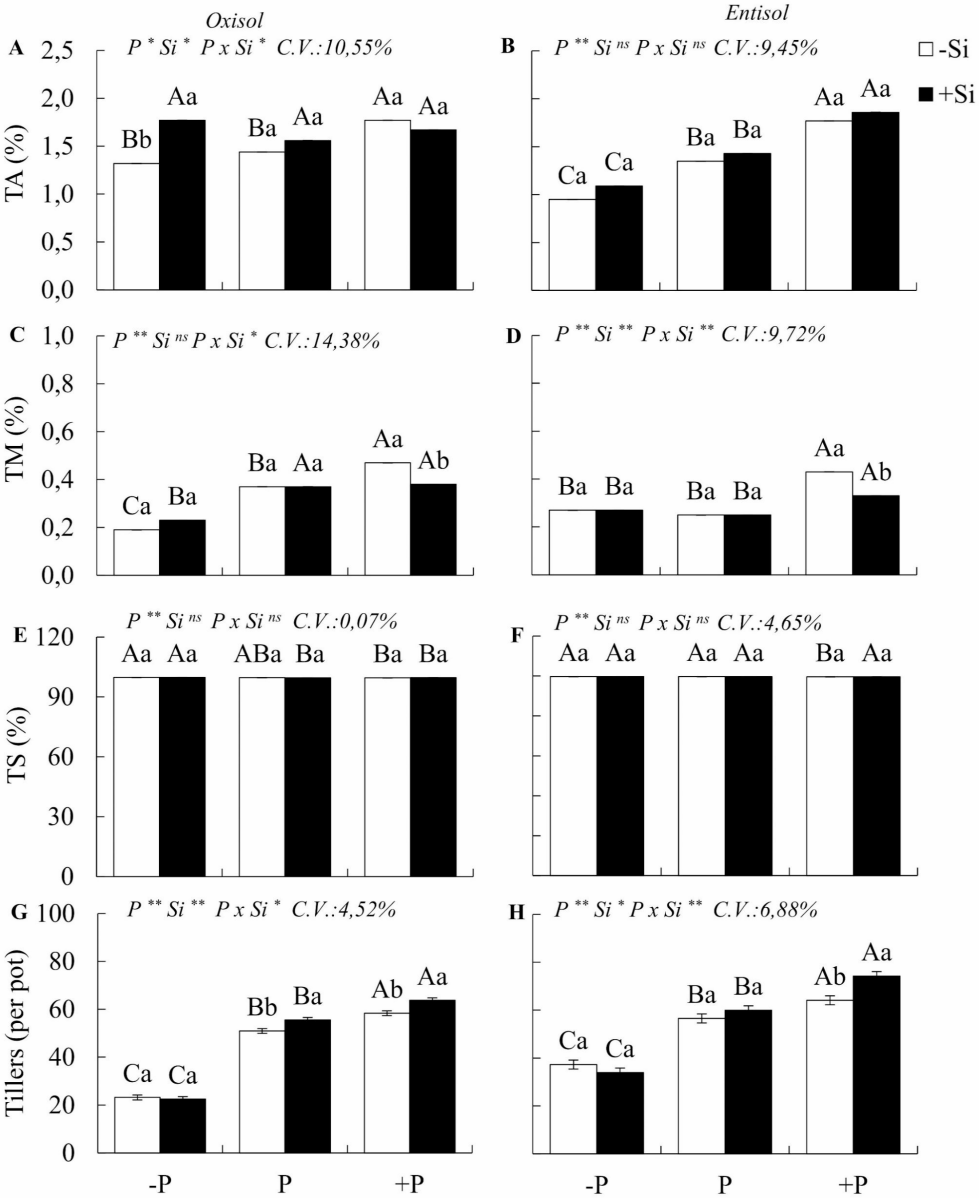
Tiller appearance rate (TA), tiller mortality rate (TM), tiller survival rate (TS), and number of tillers per pot of Zuri guinea grass under different phosphorus levels: deficiency (-P), sufficiency (P), and excess (+ P) in Oxisol (**A, C, E**, and **G**) and Entisol (**B, D, F**, and **H**) in the absence (-Si) and presence of silicon (+ Si, 1.5 mmol L^− 1^). Ns, *, ** denote the F test results as not significant and significant at 5 and 1%, respectively. Nutritional states are compared using uppercase letters, and the effect of Si is indicated with lowercase letters (Tukey test, p < 0.05). Bars represent the standard error of the experiment mean

A significant interaction PxSi (p < 0.05) was observed for most of the studied morphogenetic and structural variables (Fig. 2a-j), except for leaf elongation in Oxisol and stem diameter and leaf appearance rate in Entisol. In Oxisol, P deficiency compared to the adequate state led to an increase in leaf appearance rate and the number of live leaves per tiller (Figs. 1e and 2a). It also resulted in increased stem elongation and diameter compared to plants under P sufficiency (Fig. 2 g and 1i). Additionally, phosphorus deficiency reduced the tiller mortality rate in the Oxisol (Fig. 2c). In Entisol, P deficiency decreased the tiller appearance rate (Fig. 1b), and the number of tillers per pot in the forage, both in Oxisol (55%) and Entisol (35%) (Fig. 3a-h).

In contrast, compared to P sufficiency, excess P increased the rate of leaf appearance and leaf elongation while decreasing the number of live leaves per tiller in both soils (Fig. 2a-f). Stem elongation increased in plants under P excess in Oxisol. However, stem diameter decreased in both soils (Fig. 3 g-j). Excess P also raised the tiller appearance rate in both soils, leading to higher tiller numbers. Nevertheless, it also increased the tiller mortality rate in Oxisol and Entisol (Fig. 3a-h).

A significant interaction between P and Si was observed for the variables leaf appearance, number of leaves per tiller, and stem elongation in both soils, and leaf elongation in Entisol and stem diameter in Oxisol (Fig. 2a-j). In Oxisol, under P deficiency, the presence of Si, compared to its absence, resulted in a decrease in the number of live leaves per tiller (Fig. 2e) and an increase in the tiller appearance rate, which did not occur in Entisol (Fig. 3a, b). In Entisol, Si application to forage under P deficiency increased leaf appearance and elongation rates, decreased stem elongation, and increased stem diameter (Fig. 2b, d, h, j).

Under excess P application in Oxisol, the presence of Si, compared to its non-application, led to a decrease in the leaf appearance rate and stem elongation (Fig. 2a, g). Furthermore, it increased the number of live leaves per tiller and stem diameter (Fig. 2e, i). In Entisol with excess P, plants that received Si showed a decrease in appearance and leaf elongation rates, as well as stem elongation (Fig. 2b, c, g), and an increase in the number of live leaves and stem diameter (Fig. 2f, i).

In both soils, Si fertilization in plants under excess P resulted in a decrease in the tiller mortality rate and an increase in the number of tillers per pot, along with an increase in the tiller survival rate in Entisol (Fig. 3a-h). In P sufficiency in Oxisol, the presence of Si increased the number of live leaves per tiller (Fig. 2e) and the number of tillers per pot (Fig. 3 g). Meanwhile, in Entisol, the plants with sufficient P showed a decrease in leaf and stem elongation rates (Fig. 2d, g).

### Morphological composition and dry matter production

**Fig. 4:**
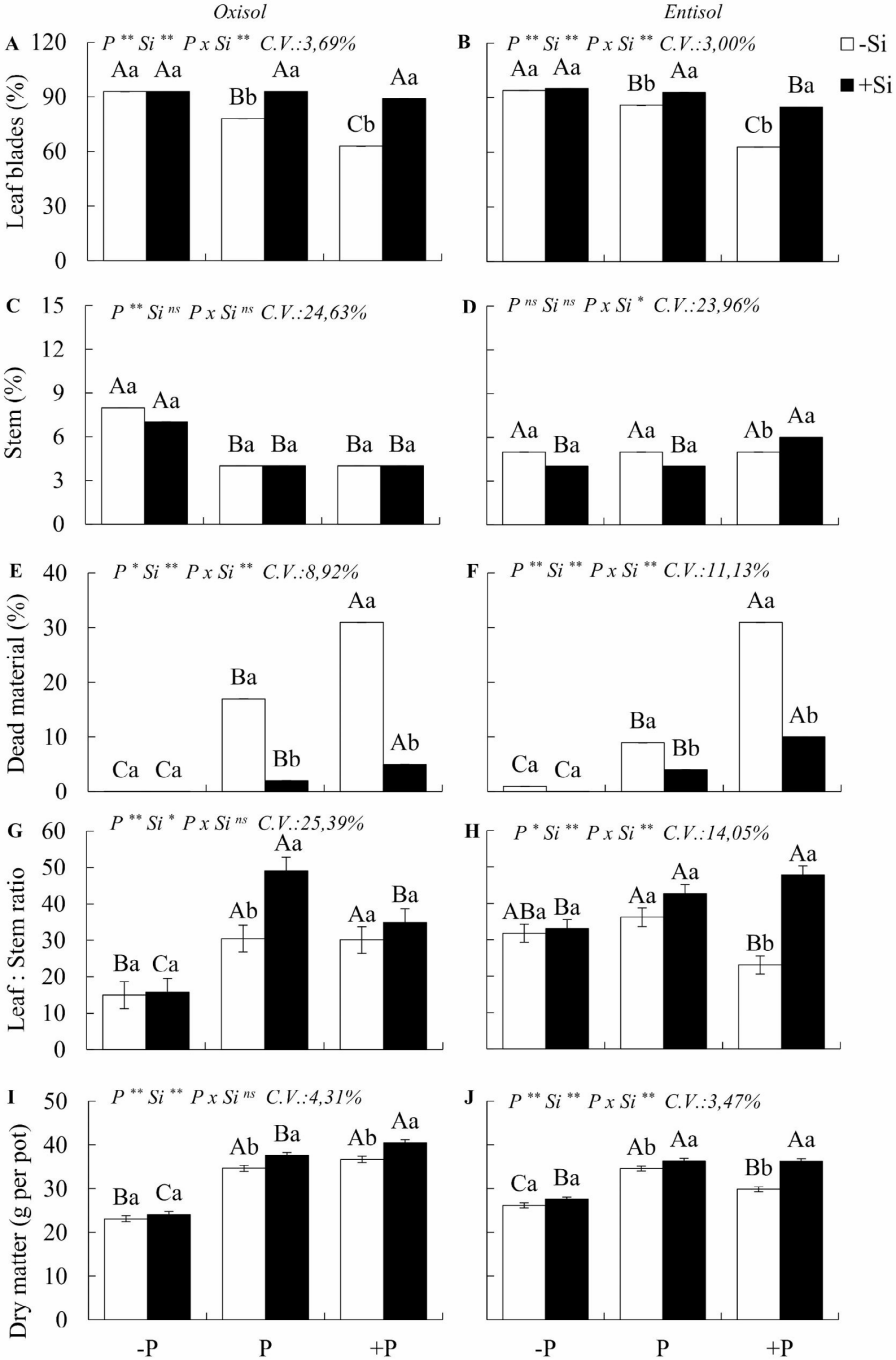
Percentage of leaf blade, stem, dead material, mass leaf:stem ratio, and dry matter yield of Zuri guinea grass under different phosphorus levels: deficiency (-P), sufficiency (P), and excess (+ P) in Oxisol (**A, C, E, G**, and **I**) and Entisol (**B, D, F, H**, and **J**) in the absence (-Si) and presence of silicon (+ Si, 1.5 mmol L^− 1^). Ns, *, ** denote the F test results as not significant and significant at 5 and 1%, respectively. Nutritional states are compared using uppercase letters, and the effect of Si is indicated with lowercase letters (Tukey test, p < 0.05). Bars represent the standard error of the experiment mean

A significant interaction PxSi (p < 0.05) was observed for the morphological variables, specifically the percentage of the leaf blade and dead material in both soils, and for the stem percentage in Entisol, but not for dry matter yield (Fig. 4a-j).

When compared to P sufficiency, *M. maximus* plants grown in Oxisol under P deficiency exhibited an increase in the green leaf blade (19%) and stem (100%) percentages, along with a decrease in the leaf:stem ratio (49%) and dead material (100%). This led to a notable 39% decrease in DM yield (Fig. 4a, c, e, g, i). Similarly, in Entisol, when compared to P-sufficient plants, those subjected to P deficiency showed an increase in the leaf blade (9%) and a decrease in dead material, resulting in a 20% reduction in DM yield (Fig. 4b, d, f, h, j). However, the presence of Si had no significant effect on the morphological variables and DM yield in plants under P deficiency in the studied soils.

**Fig. 5:**
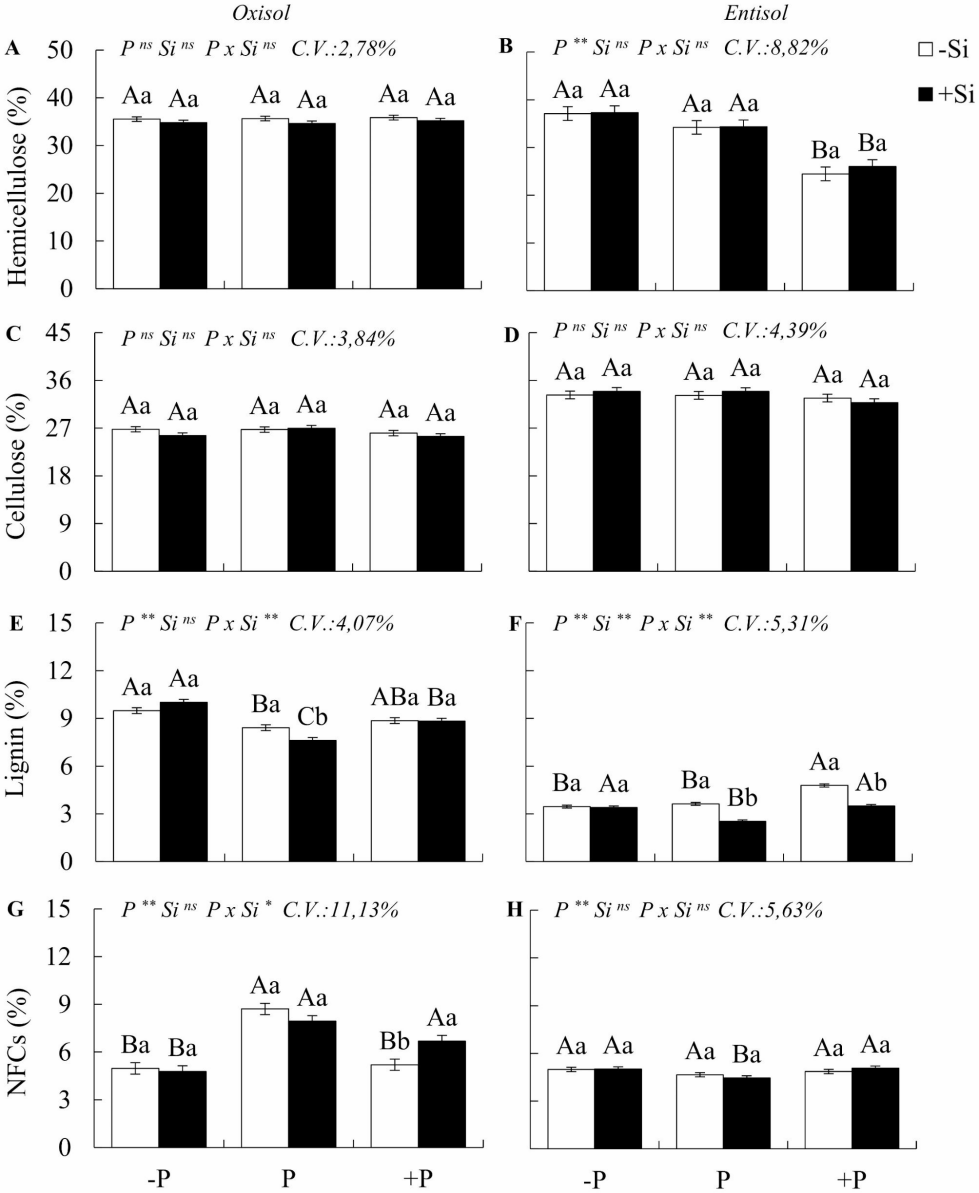
Percentage of hemicellulose, cellulose, lignin, and non-fiber carbohydrates (NFCs) in the dry matter of Zuri guinea grass cultivated during six regrowth cycles under different phosphorus levels: deficiency (-P), sufficiency (P), and excess (+ P) in Oxisol (**A, C, E**, and **G**) and Entisol (**B, D, F**, and **H**) in the absence (-Si) and presence of silicon (+ Si, 1.5 mmol L^− 1^). Ns, *, ** denote the F test results as not significant and significant at 5 and 1%, respectively. Nutritional states are compared using uppercase letters, and the effect of Si is indicated with lowercase letters (Tukey test, p < 0.05). Bars represent the standard error of the experiment mean

Cultivation under excess P without Si, in contrast to P sufficiency, resulted in a decrease in the green leaf blade (19% and 27%) and an increase in dead material (45% and 71%) in Oxisol and Entisol, respectively. It did not significantly influence the stem fraction of the plants but did increase the leaf:stem ratio in both soils (Fig. 4a-h). In Entisol, excess P led to a 14% reduction in final DM yield, which was not observed in Oxisol (Fig. 5i, j).

*M. maximus* plants under P excess that received SiO_2_ nanoparticulate experienced an increase in the leaf blade (29% and 26%) and the mass leaf:stem ratio, as well as a decrease in dead material (84% and 67%) in Oxisol and Entisol, respectively (Fig. 4a-f). Furthermore, in plants with P sufficiency and Si supplementation compared to those without Si, there was an increase in the leaf blade (16% and 7%) and the mass leaf:stem ratio and a decrease in dead material (88% and 55%) in Oxisol and Entisol, respectively. Fertilization with Si increased the total DM yield of forage in both P excess (9% and 18%) and P sufficiency (8% and 5%) in Oxisol and Entisol, respectively (Fig. 4i, j).

### Chemical composition of forage

**Fig. 6:**
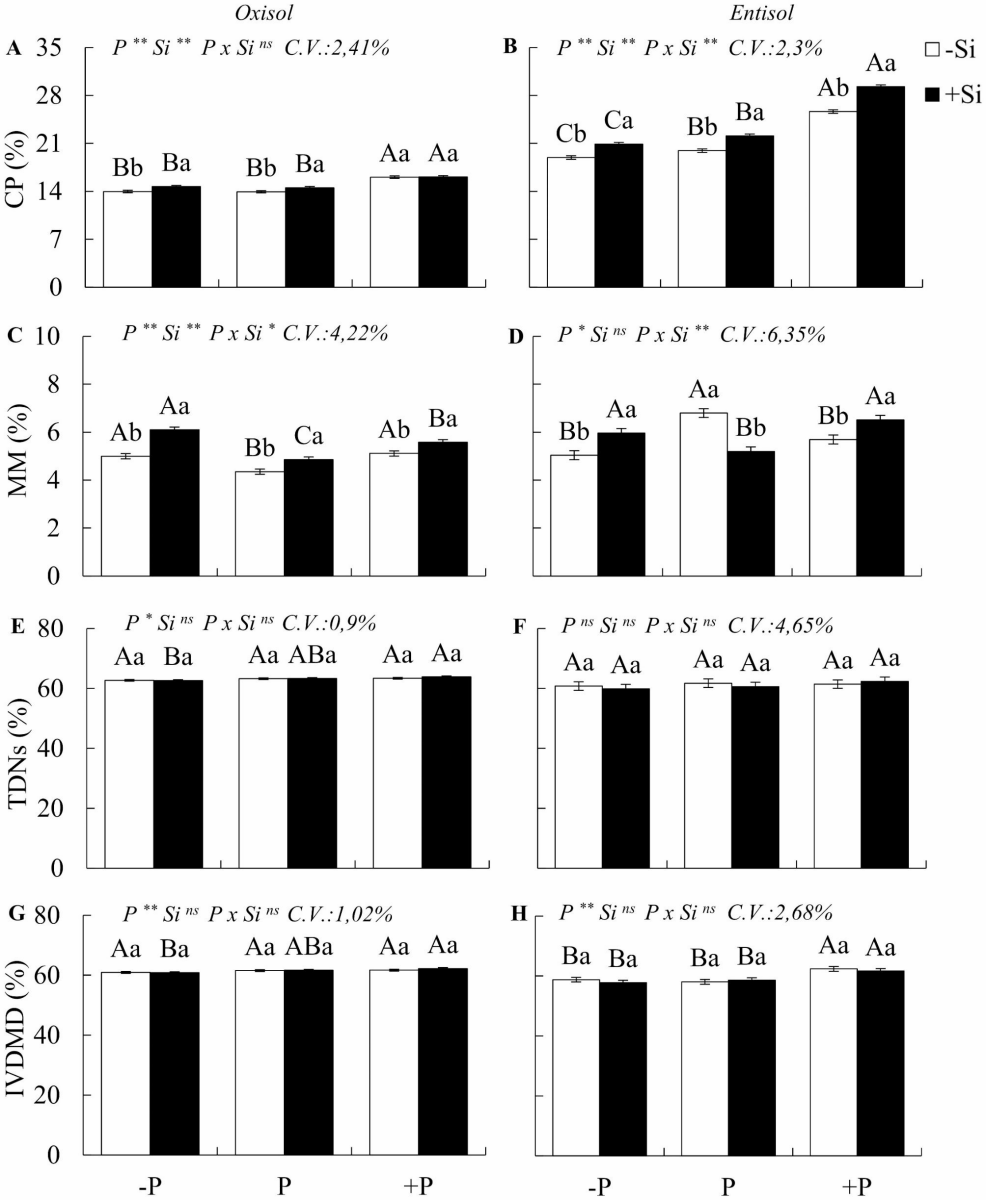
Percentage of crude protein (CP), mineral matter (MM), total digestible nutrients (TDNs), and in vitro dry matter digestibility (IVDMD) of Zuri guinea grass, cultivated during six regrowth cycles under different phosphorus levels: deficiency (-P), sufficiency (P), and excess (+ P) in Oxisol (**A, C, E**, and **G**) and Entisol (**B, D, F**, and **H**) in the absence (-Si) and presence of silicon (+ Si, 1.5 mmol L^− 1^). Ns, *, ** denote the F test results as not significant and significant at 5 and 1%, respectively. Nutritional states are compared using uppercase letters, and the effect of Si is indicated with lowercase letters (Tukey test, p < 0.05). Bars represent the standard error of the experiment mean

The results concerning variables related to chemical composition, fiber, and DM digestibility revealed a significant interaction PxSi (p < 0.05), but only for the percentage of lignin and mineral matter in both soils, and for non-fiber carbohydrates in Oxisol (Figs. 5a-h and 6a-h).

Under P deficiency in Oxisol, when compared to plants with adequate P status, *M. maximus* exhibited an increase in the percentage of lignin, a decrease in non-fiber carbohydrates, and an increase in mineral matter, with no significant effect on the CP content or DM digestibility (Figs. 5 and 6a, c, e and g). Conversely, in Entisol, deficiency did not significantly influence fiber composition or the percentage of non-fiber carbohydrates. However, it resulted in a 5.1% decrease in CP content and a reduction in mineral matter (Figs. 5 and 6b, d, f and h).

Under P excess, as compared to Oxisol sufficiency, the plants displayed a decrease in non-fiber carbohydrates and an increase in CP (15.4%) and mineral matter (Figs. 5g and 6a and c). In contrast, under Entisol conditions, excess P led to a decrease in hemicellulose (28.5%) and mineral matter, coupled with an increase in lignin (1.1%), CP (28.6%), and DM digestibility (Figs. 5b and f and 6b, d and h).

With the addition of Si, plants experiencing P deficiency in relation to nutrient sufficiency exhibited increased CP content (4.9% and 9.4% in Oxisol and Entisol, respectively) and mineral matter (Fig. 6a-d), with no significant impact on fiber composition or digestibility. In cases of excess P in plants cultivated in Oxisol, Si fertilization resulted in increased non-fiber carbohydrates and mineral matter. In Entisol, there was a decrease in lignin (1.3%) and an increase in CP content (12.4%), as well as mineral matter. The presence of Si in conditions of P sufficiency, as opposed to its absence, led to a reduction in the percentage of lignin in both soils (1.0%). Furthermore, it increased the CP content in Oxisol (4.1%) and Entisol (9.7%) and elevated the mineral matter in Oxisol. Regardless of the P nutritional status, Si fertilization had no impact on TDN percentage or in digestibility.

**Fig. 7:**
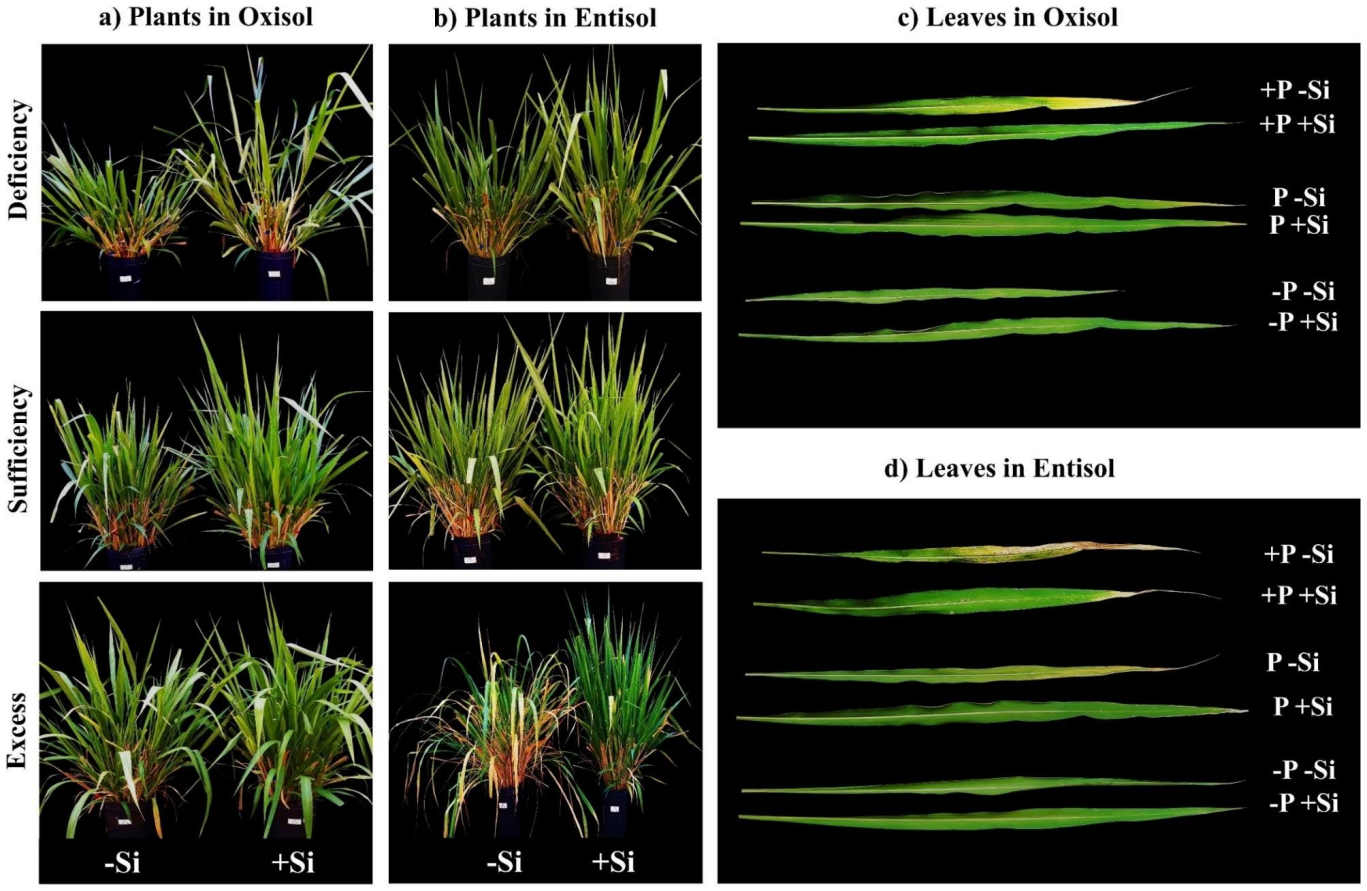
Plant phenotype and visual aspects of the leaf blades of *M. maximus* cv. Zuri grown under conditions of phosphorus deficiency, sufficiency, and toxicity, combined with the absence (-Si) or presence of Si nanoparticles (+ Si, 1.5 mmol L^− 1^) in Oxisol (**a, c**) and Entisol (**b, d**) at the end of the fifth regrowth cycle

## Discussion

### P imbalances and Si absorption

P deficiency and excess were clear in both soils, confirmed by the low and high content in the plant, respectively, in relation to plants in an adequate state of the nutrient (Fig. 1a, b). The low levels of P available in weathered soils led to the occurrence of deficiency, and the dose applied to cause excess P increased its content in the plant in both soils, reaching 18.8 and 29.9 g kg^− 1^ in cultivated plants in Oxisol and Entisol, respectively. The same dose of P caused a greater increase in the nutrient content in the plant in Entisol, due to the greater availability of phosphate in this soil compared to Oxisol, which has a higher clay content [2]. Thus, in sandy soil the risk of P toxicity in plants under excessive fertilization is greater than in clayey soil.

Nanosilica fertigation was efficient in increasing the Si content in plants compared to non-application. This increase in Si content was more prominent in P deficiency plants in both soils, and also in excess P in Entisol, revealing that Si is an element required by plants subjected to P imbalances, in a similar way to abiotic stresses [46, 47]. It is pertinent to admit that Si can reduce the risk of P toxicity in soils under nutrient excess, given its action in decreased P content of plants in both soils. The Si mechanisms involved in this regulation still need to be elucidated, especially for its action as a regulator of the expression of P transporters [48].

### Deficiency and excess of P without the use of Si in pasture morphogenesis and yield

While the effects of phosphorus (P) deficiency on grasses have been well-documented [1, 3, 49], there is relatively limited research focusing on the morphogenic processes of grasses used in pastures under P deficiency [50, 51]. This is a notable gap in the literature because understanding how nutritional disorders of P affect these plants and how they respond morphogenetically to such stress is crucial.

In our study, we observed several strategies employed by *M. maximus* to cope with P deficiency, which was evident in plants cultivated in both soils studied. Compared to plants under P sufficiency, those experiencing P deficiency increased the number of live leaves per tiller without decreasing leaf elongation rates. However, this strategy came at the cost of a significant reduction in tiller appearance rate and the overall number of tillers, resulting in diminished biomass yield.

The growth and tillering dynamics of forage grasses involve morphogenic processes crucial for both forage yield and pasture longevity [11]. Our findings shed light on the morphogenetic strategies employed by M. maximus to mitigate the effects of P deficiency in both clayey (Oxisol) and sandy (Entisol) soils. In Entisol, the morphophysiological changes in *M. maximus* under P deficiency manifested as a reduced tiller appearance rate due to decreased cell division and expansion. This outcome aligns with observations made in perennial *Lollium* grasses [52].

The decrease in tillering observed under macronutrient deficiency occurred in both soils. However, in Oxisol, plants responded by increasing appearance rates and leaf elongation. This difference may be attributed to a more severe degree of P deficiency in the plants grown in Oxisol, possibly prompting a more efficient utilization of the available nutrient. It is known that under P deficiency, plants can allocate more P to essential metabolic processes [53, 54]. Additionally, while each tiller has a genetically predetermined number of leaves [55], leaf elongation is a trait influenced by genotype-environment interactions in forage grasses [56], as we observed in relation to P.

The reduction in tillering had a severe impact on forage yield, a common occurrence in grasses under P deficiency [12, 54, 57]. The reduced yield of nutritionally deficient plants was anticipated, considering they were grown in weathered soils with low available P content (12 and 2 mg dm^− 3^ in Oxisol and Entisol, respectively). This limitation compromises cell division and hinders net photosynthesis rates [7, 49].

Interestingly, the decrease in DM yield due to P deficiency was 18% more pronounced in Oxisol than in Entisol. This difference may be explained by the fact that plants in Entisol likely utilized the small amount of added P (4 mg dm^− 3^) more efficiently to enable minimal growth, which accounted for only 2% of the recommended dose [27]. However, this was sufficient to mitigate the severity of P deficiency to a certain extent in Entisol, which has a lower phosphate adsorption capacity [2, 49, 51].

Plants cultivated under excess P exhibited impairments, possibly stemming from disrupted cellular phosphate (Pi) homeostasis. This imbalance affects essential P-related functions in plants, leading to decreased photosynthesis and biomass yield [5, 8]. Our study suggests, for the first time, that an excess of P in the soil disrupts the balance between C, N, and P in grasses, subsequently hindering the efficient utilization of these nutrients [58]. However, there is still a limited number of studies describing the effects of excess P on grasses, even in nutrient solutions [59]. Thus, our study may represent one of the first attempts to identify morphogenic responses of forage grasses to excess P in different soils.

Our research provides a deeper understanding of the morphogenic mechanisms of *M. maximus* and highlights the plant’s response strategies when exposed to excess P compared to plants with adequate P levels in both soils. Surprisingly, we found that the forage, when subjected to toxic P levels, increased leaf appearance and elongation rates but simultaneously maintained a reduced number of live leaves on each tiller. Consequently, this led to an increase in tiller appearance and numbers, albeit with smaller diameters, higher tiller mortality rates, lower percentages of live leaf blades, higher amounts of dead material, and ultimately reduced dry matter yield.

In contrary to P deficiency, excess P accelerates the growth dynamics of M. maximus. However, the higher tiller mortality observed in plants under excess P in both soils may result not only from the increased stimulus to cell division processes due to P excess but also from the synergistic effects between P and N. Under P excess, plants can absorb higher quantities of N, contributing to increased tissue renewal [60, 61]. [54] also noted that tissue renewal processes in Massai grass (*M. maximus* x *M. infestum*) are accelerated with increasing phosphorus doses, even in the absence of toxic P levels.

Early leaf senescence and associated losses were observed in plants grown under both P deficiency and excess. However, excess P only significantly impacted total DM yield in Entisol, which aligns with the higher phosphate availability in sandy soils [2]. Moreover, in soils rich in oxidic clays like Oxisol, the availability of P decreases with prolonged exposure to adsorption sites [62]. Thus, the implications of excess P in pastures are even more concerning when considering sandy soils, partially supporting our initial hypothesis. Although both P imbalances altered the growth and tillering of plants in both soils, only the excess of P reduced the DM yield of Zuri grass when cultivated in sandy soil.

### Deficiency and excess of P without the use of Si in the morphological, chemical composition and nutritional value of forage

Our observations indicate that P imbalances also induce changes in the morphogenic and structural characteristics, chemical composition, and nutritional value of *M. maximus* grass. However, although P deficiency resulted in increased hemicellulose and decreased protein in plants grown in one soil type (Entisol) and increased lignin and mineral content, while decreasing non-fibrous carbohydrates in another soil type (Oxisol), these changes did not significantly impact the total digestible nutrients (TDN) and in vitro dry matter digestibility (IVDMD) of the grasses cultivated in both soils. The notable increase in lignin content in P-deficient plants grown in Oxisol is a known effect observed in plants exposed to abiotic and biotic stress as part of their natural defense mechanisms [63]. However, it is a costly strategy in terms of energy expenditure for the plant [64].

The lack of an effect of P deficiency on digestibility can be attributed to the fact that these plants maintained a greater number of young leaves. This occurred because plants in this condition typically had fewer tillers, which prompted an increase in leaf blade and stem yield in response to stress. Furthermore, plants under P deficiency exhibited delayed leaf senescence, which had a positive impact on forage digestibility.

In the case of plants subjected to P deficiency in Entisol, one notable effect was the reduction in the plant’s crude protein (CP) content. This CP fraction includes protein nitrogen (true protein) and non-protein nitrogen, such as amino acids and amines, which are crucial for animal nutrition [65]. The decrease in protein levels in these plants was attributed to the fact that P is essential for ribonucleic acid (RNA), a requirement for the translation process in protein synthesis [7]. Moreover, P-deficient plants experience reduced nitrogen (N) uptake, which is vital for protein formation [66, 67].

Another effect of P deficiency in plants grown in Oxisol was the increase in mineral matter content. This increase may have resulted from higher elemental concentrations in the dry matter, given that these plants exhibited less growth and biomass yield compared to adequately nourished plants (Fig. 7a, b). Alternatively, the increase in mineral matter could be attributed to the uptake of residual silicon (Si) from the soil. Stressed plants often release exudates into the rhizosphere, promoting the dissolution of phytoliths within organic matter [68], thus releasing Si. In summary, pastures of *M. maximus* under P deficiency produced less dry matter compared to well-fertilized pastures, and while they exhibited lower protein content, it did not negatively affect digestibility.

Another significant nutritional concern arises with the issue of excess P in the grass. However, the effects of excess P on the nutritional value of the plant had remained largely unexplored until our study. We observed that excess P initially decreased the mass leaf:stem ratio due to the reduced percentage of live leaf blades, a phenomenon previously described by [10]. These visual symptoms, such as chlorosis and necrotic spots on the leaves (Fig. 7c, d), mark the onset of P toxicity in this species, which eventually progresses to complete leaf necrosis. These symptoms result from biochemical disruptions in plant tissues caused by excessive P, including photosynthesis inhibition and increased lipid peroxidation [8], along with accelerated senescence and reduced photosynthetically active areas. This ultimately leads to decreased dry matter accumulation [9, 69], resulting in biomass loss, as grazing animals typically select green leaf blades with greater nutritional value [70, 71].

That excess P, similar to P deficiency, resulted in a higher lignin content in *M. maximus*, serving as a strategy to mitigate nutritional stress, consistent with findings from [72]. This underscores the variability in fiber lignin content associated with nutrient inputs [14]. However, even with increased lignin content, we visually noted that plants under P stress, especially those under P excess, did not attain a phenotype resembling well-nourished P plants, which typically exhibit well-developed, green leaf blades, characteristic of most forage grasses (Fig. 7a-d).

Although higher lignin content may affect fiber digestibility by ruminants [63, 73], our study did not find any significant impact on dry matter digestibility despite P imbalances. This can be explained by the fact that the lignin primarily consisted of non-core lignin, mainly composed of low-molecular-weight phenolic compounds released from the cell wall through hydrolysis. These compounds are typically represented by ester-linked p-hydroxycinnamic acids, as opposed to core lignin, which features highly condensed cell wall phenylpropanoid polymers that resist microbial degradation in the rumen [74, 75].

The increase in CP content observed in plants under excess P in Entisol can be attributed to P’s essential role in protein synthesis. However, it is crucial to note that this may also be related to the fact that P stimulates N uptake [60]. Nevertheless, the higher protein content in plants under P excess results in nutrient loss, as these nutrients cannot be efficiently redistributed within the plant for metabolic processes or used by grazing animals that typically avoid consuming dead leaves.

Considering the results discussed, our study partially confirms the second hypothesis. While P imbalances did indeed alter the chemical composition of the plant, they did not affect forage digestibility. However, the significant reduction in biomass yield caused by excess P and early leaf senescence ultimately compromises the availability of high-quality pasture [70].

### Benefits of Si in the morphogenesis and production of plants with deficiency, sufficiency, and excess P

This study has provided a comprehensive understanding of the detrimental effects of P imbalances on forage, which significantly impact extensive cultivation areas of these species worldwide. Given these findings, there is an urgent necessity to develop strategies for mitigating this damage without harming the environment. The use of Si can be synergistic with P uptake by plants as silicates compete with phosphate for adsorption sites, which increases available phosphate in the soil [19, 76]. However, the effects of Si on the morphogenesis of forage plants, especially with the use of innovative sources of the element, such as nanosilica, are unknown, and it is opportune to further research on this topic, which motivated this study.

The benefits of silicon in the morphogenesis of P-deficient forage were somewhat limited, as it only increased the tiller appearance rate in plants grown in Oxisol and resulted in an increase in the appearance rate, leaf elongation, and stem diameter in plants grown in Entisol. However, these improvements in morphogenesis induced by silicon under P deficiency were insufficient to significantly enhance plant dry mass yield in the two soils studied. This may be attributed to the fact that there was no significant increase in the number of tillers, which represents the basic unit of grass yield [10].

In hydroponic studies, silicon has been shown to alleviate P deficiency in wheat and rice [77, 78]. However, these studies involve more rigorous control of P levels in the culture medium (nutrient solution), which generates a more severe deficiency in plants compared to soil cultivation. There are indications that the beneficial effects of silicon are more pronounced under severe stress conditions [79]. Furthermore, advanced forage regrowth cycles may exacerbate P deficiency in the soil due to nutrient export in the biomass, potentially enhancing the benefits of silicon in plants. Nonetheless, this remains a subject for future research [51].

Concerning the role of silicon in plants with excess P, this is an uncharted territory in this species, as P excess is less widespread compared to P deficiency. Nevertheless, it is essential to initiate research in this area. Our study revealed that silicon triggers certain morphogenic processes in the plant to counteract the damage caused by excess P in *M. maximus*, which was observed in plants cultivated in both soils. However, this was accompanied by a decrease in the appearance rate of new leaves due to the control of tissue flow within the plant. This led to an increase in the number of live leaves per tiller and a decrease in stem elongation rate, ultimately favoring greater tiller numbers and thicker tiller diameters. These improvements in plant growth and structure translated into an increase in plant dry mass yield in Entisol.

However, it should be noted that in plants grown in Oxisol with the P dosage used, it was not possible to induce P toxicity due to this soil’s high capacity for P adsorption. Therefore, since P toxicity did not manifest in plants grown in this soil, it was not feasible to investigate the mitigating effects of silicon in such a scenario. Nevertheless, the risk of P toxicity in Oxisol remains relatively low, and further research using higher P doses (> 600 mg dm^− 3^ of P equivalent to 1200 kg ha^− 1^ of P) may be required, as Oxisol’s maximum P adsorption capacity is approximately 575 mg dm^− 3^ and may vary depending on other soil properties, such as organic matter content [2].

Considering these results obtained in plants cultivated in Entisol, it is plausible that mitigating the damage caused by excess P in *M. maximus* may have similar benefits in other species [76, 80]. In a study involving rice plants cultivated in a nutrient solution with excess P, silicon was found to downregulate the gene expression of phosphate transporters, thereby reducing phosphate uptake by the plant [48]. This mechanism may be one of the ways silicon mitigates P excess in grasses and warrants further investigation.

Studies involving silicon typically focus on stress conditions, with the prevailing belief that silicon benefits only stressed crops, offering no advantages to non-stressed plants [79]. However, our study evaluated the effects of silicon on the morphogenesis of plants under P sufficiency, i.e., plants without stress. We observed an increase in the number of live leaves per tiller and the number of tillers in plants grown in Oxisol, as well as a decrease in stem elongation in plants grown in Entisol.

These positive effects of Si reflected an increase in DM production in *M. maximus* in both soils, possibly due to different Si mechanisms reported in grasses, such as the promotion of adequate nutritional homeostasis to provide an increase in the efficiency of N and P use [20, 47, 81, 82], even in plants without stress [83]. Our results corroborate the fact that Si can influence the morphology of plants [23], mainly in Si-accumulating species [84], and can improve the productivity of non-stressed plants and mitigate nutritional stress.

In conclusion, it was clear that the utility of silicon for *M. maximus* cultivation depends on the presence or absence of stress, the type of stress, and the soil class. Silicon can offer benefits to *M. maximus* in regions worldwide, whether it covers areas without nutritional stress in sandy or clayey soils or addresses areas with excess P, particularly in sandy soils, thus making it a valuable tool for sustainable agricultural yield [80]. Furthermore, it can be used in areas with excess P, especially in sandy soils, enabling its use in agriculture. This discovery confirms the important role of Si as a tool for more sustainable agricultural production [46].

### Benefits of Si on the morphological and chemical composition and nutritional value of forage

An issue of concern within the scientific community regarding silicon’s presence in forage is its potential role as an anti-nutritional element in animal feed [85]. Furthermore, it remains unclear whether silicon has a beneficial or detrimental effect on forage quality under various cultivation conditions, including areas with ample soil fertility or those with phosphorus (P) deficiency or excess. This is a pressing concern because, as we have observed, silicon may significantly influence morphogenesis and plant growth. However, these advantages would be futile if there were substantial losses in forage quality, ultimately affecting the performance of animals consuming such forage.

In an effort to address this question, our study demonstrated that the use of nanosilica in P-deficient plants did not result in alterations to the morphological or fiber composition of *M. maximus*. Instead, it led to increased crude protein (CP) and mineral matter content. This increase in protein content can be attributed to silicon’s role in enhancing the uptake of both P [78] and nitrogen (N) [86], both of which are integral to protein synthesis in plants. This finding is of paramount importance due to the significance of protein for the ruminal microbiota’s activity [14].

The increase in mineral matter in the presence of silicon reflects the uptake of this beneficial element by stressed plants [19, 77]. Notably, utilizing silicon in these plants did not hinder forage digestibility, possibly because silicon did not induce lignification. Nevertheless, it is worth noting that P-deficient wheat subjected to silicon exhibited enhanced lignin synthesis [87], indicating that species may adopt varying strategies for mitigating stress, involving silicon in cell wall composition and/or activating enzymes related to lignin synthesis, which should be elucidated in future studies.

This study unequivocally demonstrates that the introduction of silicon into M. maximus, even under P deficiency, does not compromise the forage’s nutritional value. This is a highly practical revelation, as most pastures cultivated in tropical regions contend with P-deficient soils [1] and could immensely benefit from the sustainable application of this element. In regions with excess P, incorporating silicon also yielded promising outcomes for M. maximus. This resulted in improved morphological composition of the plant, with reduced dead material and increased leaf blade fractions in both soil types. Additionally, it’s noteworthy that there was a decrease in the tiller mortality rate—a pivotal factor for pasture persistence [11]. Importantly, it should be highlighted that, in line with observations in other crops subjected to excess P [88, 89], silicon supplementation in M. maximus grown under P excess led to reduced lignin content in the fiber and increased CP and mineral matter content, especially in plants cultivated in Entisol.

While some advantages of lignin have been reported for stressed plants, such as enhanced leaf architecture [90] and the antioxidative action of phenolic precursors against reactive oxygen species [91], it is not considered a favorable strategy for forage plants since lignin is an anti-nutritional component for ruminants [75]. Our study confirmed that in *M. maximus*, silicon does not promote lignin synthesis, even in plants exposed to excess P. This may be attributed to the plant’s utilization of silicon at a low energy cost for cell wall composition, effectively substituting lignin’s role in plant tissue support, ultimately leading to reduced lignin synthesis [92].

Therefore, the significant contribution of nanosílica fertilization to the improvement of morphological and chemical composition in forage grasses was evidenced, even under excess P. As such, our third hypothesis was partially confirmed, as nanosilica fertilization mitigated the stress associated with excess P without compromising forage quality. Moreover, it is evident that silicon application in plants with sufficient P nutrition led to decreased lignin content, which may enhance fiber digestibility [63], and joins other promising results in grasses without nutritional stress [92]. Additionally, our study revealed that silicon supplementation increased protein content in plants with optimal P nutrition, corroborating findings observed in Zuri (*M. maximus*) and Ipyporã *(Urochloa ruziziensis* x *U. brizantha*) grasses following silicate fertilization [28].

However, plants tend to absorb silicon in greater quantities when subjected to stress [93]. Consequently, the decrease in forage lignin content under P sufficiency likely stemmed from a slight increase in silicon content. Similar findings were reported in rice plants and sorghum without stress, indicating a negative correlation between silicon accumulation and lignin synthesis [88, 89], a relationship consistent with our observations under P sufficiency. Further investigations into the connection between silicon fertilization and lignin biosynthesis in grasses are imperative, including studies involving properly nourished plants, as results appear to be contingent on the crop and growing conditions.

In conclusion, these results underscore the advantages of silicon fertilization in forage grasses under P stress or with adequate nutrition. This practice optimizes the integrated soil-forage-animal system, resulting in the improved formation of pastures, recognized as the most economically efficient food source for animal yield according to [14]. Thus, the use of Si can help recover the potential productive of pastures, with a more efficient use of productive resources, improving the productivity and sustainability of pasture production.

## Conclusions and future perspectives

In summary, this study provides novel insights into the underlying causes of growth reductions in *M. maximus* cv. Zuri when subjected to both P deficiency and excess P in two tropical soils. Furthermore, we elucidate the plant’s morphogenic and structural adaptive strategies to mitigate these stresses, all while maintaining forage quality.

Moreover, we demonstrate that the application of SiO_2_ nanoparticles fosters enhanced equilibrium within the plant’s growth and senescence processes, mitigating biomass yield loss, particularly in plants cultivated under excess P in sandy soil. Additionally, SiO_2_ nanoparticle fertilization increases yields under P sufficiency in soils with varying textures without compromising digestibility.

Future research endeavors should delve into Si-specific mechanisms, employing metabolomic approaches to better understand Si’s role in alleviating damage caused by P excess in grasses and optimizing yields in non-stressed plants. This investigation should also explore Si’s impact on reducing plant senescence, which was notably observed in this study. Furthermore, examining Si’s potential in ameliorating P deficiency in forage grasses warrants exploration, given the observed potential to enhance the growth and protein content of deficient plants, including testing with different Si sources and doses, to find the economic viability of their use.

## Data Availability

The datasets generated and/or analysed in this study are available from the corresponding author upon reasonable request.

## Declarations

### Research involving plants

The experimental research was carried out in accordance with relevant institutional, national, and international guidelines and legislation. It does not involve an endangered species.

## Abbreviations

ADF: Acid detergent fibre
C: Carbon
CP: Crude protein
DM: Dry matter
LA: Leaf appearance rate
LE: Leaf elongation rate
MM: Mineral matter
NDF: Neutral detergent fibre
N: Nitrogen
NFCs: Nonfiber carbohydrates
P: Phosphorus
SE: Stem elongation rate
SD: Stem diameter
Si: Silicon
TA: Tiller appearance rate
TDN: Total digestible nutrients
TM: Tiller mortality rate
TS: Tiller survival rate
